# Beneficial Effects of Montelukast Against Methotrexate-Induced Liver Toxicity: A Biochemical and Histological Study

**DOI:** 10.1100/2012/987508

**Published:** 2012-04-01

**Authors:** Evren Kose, Hilal Irmak Sapmaz, Ediz Sarihan, Nigar Vardi, Yusuf Turkoz, Nihat Ekinci

**Affiliations:** ^1^Department of Anatomy, School of Medicine, İnonu University Medical Faculty, 44280 Malatya, Turkey; ^2^Department of Emergency, Inonu University, 44280 Malatya, Turkey; ^3^Department of Histology-Embryology, Inonu University, 44280 Malatya, Turkey; ^4^Department of Biochemistry, Inonu University, 44280 Malatya, Turkey

## Abstract

The effects of montelukast against methotrexate-induced liver damage were investigated. 35 Wistar albino female rats were divided into 5 groups as follows: group I: control; group II: montelukast (ML); group III: methotrexate (Mtx); group IV: montelukast treatment after methotrexate application (Mtx + ML); group V: montelukast treatment before methotrexate application (ML + Mtx). At the end of the experiment, the liver tissues of rats were removed. Malondialdehyde (MDA), myeloperoxidase (MPO), and reduced glutathione levels were determined from liver tissues. In addition, the liver tissues were examined histologically. MDA and MPO levels of Mtx group were significantly increased when compared to control group. In Mtx + ML group, these parameters were decreased as compared to Mtx group. Mtx injection exhibited major histological alterations such as eosinophilic staining and swelling of hepatocytes. The glycogen storage in hepatocytes was observed as decreased by periodic acid schiff staining in Mtx group as compared to controls. ML treatment did not completely ameliorate the lesions and milder degenerative alterations as loss of the glycogen content was still present. It was showed that montelukast treatment after methotrexate application could reduce methotrexate-induced experimental liver damage.

## 1. Introduction

Methotrexate (Mtx), a structural analogue of folic acid, is widely used as a chemotherapeutic agent for cancer treatment and for autoimmun diseases [1–3]. With the widespread use of Mtx, hepatotoxicity is the most important potential major side effect [4, 5]. It has been reported that liver damage may occur as well in particular high doses or following chronic administration of Mtx [6, 7].

Leukotrienes (LTs) are synthesized from membrane phospholipids in response to cell activation. Cysteinyl-leukotrienes (CysLTs) are produced from arachidonic acid through 5-lipoxygenase (5-LO) pathway and act on the CysLT_1_ and CysLT_2_ receptors [8]. In fact, several pathways are involved in production of reactive oxygen species (ROS), it has been reported that bioactive metabolites of LTs have a pivotal role in oxidative stress [9]. In another study, Beytur et al. [10] reported that the selective reversible CysLT_1_ receptor antagonist, montelukast (ML) (MK-0476), has significant antioxidant properties against CP-induced testicular damage. Previously, we have shown that ML treatment after Mtx application could reduce Mtx-induced renal damage [11]. Also, the protective effects of ML have previously been addressed in other models of cell damage induced by several drugs [12]. The beneficial effects of ML in various experimental models of inflammation have also been reported [13, 14].

To our knowledge, there is no report regarding the protective and therapeutic effects of ML against Mtx-induced acute liver toxicity. Therefore, the current study was designed to explore the therapeutic and protective effects of montelukast against Mtx-induced acute liver damage in rats.

## 2. Methods

### 2.1. Animals

35 Wistar albino female rats were housed in an air-conditioned room with 12-h light and dark cycles, where the temperature (22 ± 2°C) and relative humidity (65–70%) were kept constant. All experimental protocols were approved by the Inonu University, School of Medicine Animal Care and Use Committee, Malatya, Turkey.

### 2.2. Experimental Protocol

The rats were divided into 5 groups as follows: group I: control; group II (ML): control + montelukast (Notta tb 10 mg, Sanovel, Turkey, 10 mg/kg daily for 10 days p.o.); group III (Mtx): methotrexate (Methotrexate 50 mg, Koçak Farma, Turkey, single dose 20 mg/kg i.p.); group IV (Mtx + ML): methotrexate (single dose 20 mg/kg i.p.) + montelukast (10 mg/kg daily for 10 days p.o., after 3 days methotrexate injection); group V (ML + Mtx): montelukast (10 mg/kg daily for 10 days p.o.) + methotrexate (single dose 20 mg/kg i.p, after the last dose of montelukast). At 24 h after the last injection, rats in all groups were killed and the liver tissues of rats were collected for further analyses. Part of the liver tissue specimen was placed in formaldehyde solution for routine histopathological examination by light microscopy. The other part was placed in liquid nitrogen and stored at −70°C until assay for malondialdehyde (MDA), reduced glutathione (GSH), and myeloperoxidase (MPO).

### 2.3. Biochemical Analysis

The liver tissues were individually homogenized in ice-cold 0.1 M Tris-HCl buffer (pH 7.5) with a homogenizer (IKA Ultra Turrax T 25 basic, IKA Labotechnik, Staufen, Germany) at 16000 rpm for 3 min. The homogenates were used to measure the levels of MDA, GSH, and MPO. All procedures were performed at 4°C.

MDA levels were assayed spectrophotometrically at 535 and 520 nm according to the method of Uchiyama and Mihara [15]. The results are expressed as nanomoles per gram wet tissue.

GSH levels were measured using the method of Elman [16]. GSH is reacted with 5,5-dithiobis-2-nitrobenzoic acid resulting in the formation of a product which has a maximal absorbance at 410 nm. The results are expressed as nanomoles per g wet tissue.

Determination of MPO activity was carried out spectrophotometrically (T60U Spectrometer, PG Instruments Limited, AlmaPark, Wibtoft, Leicestershire, UK) using 4-aminoantipyrine/phenol that is a substrate for MPO-mediated oxidation by H_2_O_2_. The absorbance was read at 510 nm and the data were given as U/g protein [17].

### 2.4. Histological Assessment

Liver tissue was fixed in 10% formalin and was embedded in paraffin. Tissue sections were cut at 5 *μ*m, mounted on slides, stained with hematoxylineosin (H-E) for general liver structure and periodic acid schiff (PAS) to demonstrate the glycogen deposition in hepatocytes. The sections were examined by a Leica DFC 280 light microscope by a histolog unaware of the status of animals. The liver damage severity was semiquantitatively assessed as follows; hepatocytes with eosinophilic cytoplasm, hydropic degeneration (cytoplasmic vacuolization and swelling of hepatocyte), and loss of the glycogen deposition in hepatocytes. Microscopic damage was identified as absent (0), slight (1), moderate (2), and severe (3), for each criterion.

### 2.5. Statistical Analysis

The results were compared with Kruskal-Wallis variance analysis. Where differences among the groups were detected, group means were compared using the Mann-Whitney *U* test. Values of *P* < 0.05 were considered significant. All results were expressed as means ±  standard  deviation (SD).

## 3. Results

### 3.1. Biochemical Results

Table 1 summarizes the data obtained on the effects of Mtx and treatment of montelukast on tissue MDA, MPO, and GSH levels. In brief, methotrexate treatment caused an elevation of MDA and MPO productions when compared to the control group (*P* < 0.05). Montelukast application after methotrexate injection (Mtx + ML) reduced these parameters significantly, whereas treatment of montelukast before methotrexate injection (ML + Mtx) could not show any beneficial effects on MDA and MPO levels. When the GSH levels increased in Mtx group, decreased in Mtx + ML and ML + Mtx groups. But these changes are not significantly.

### 3.2. Light Microscopic Evaluations

**Figure 1:**
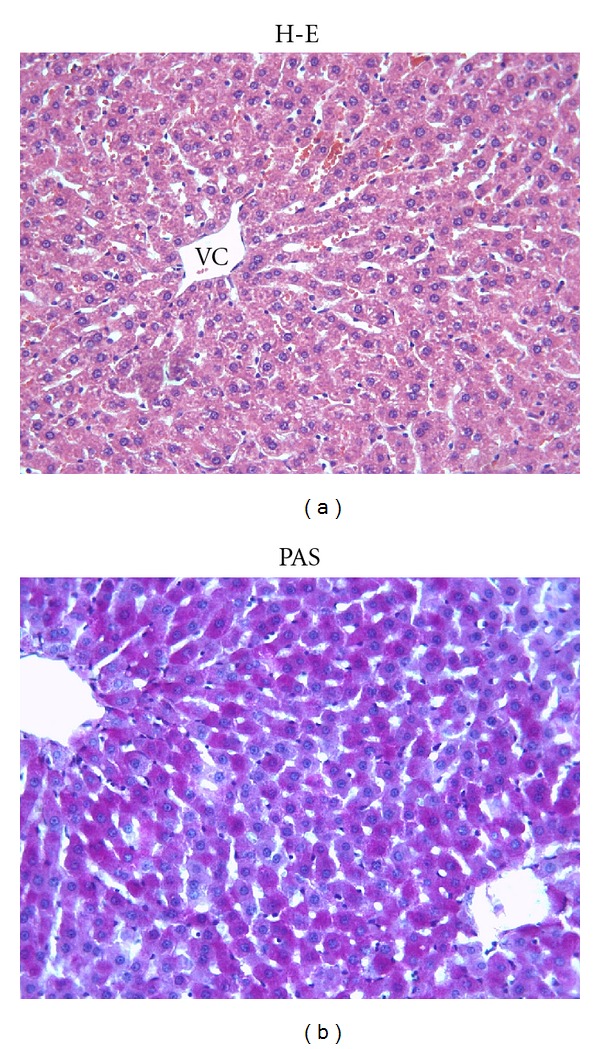
(a) Normal histological appearance of liver in the control group. VC: Vena centralis. (b) The PAS-positive reaction shows a magenta staining where glycogen is present within hepatocytes. X66.

**Figure 2:**
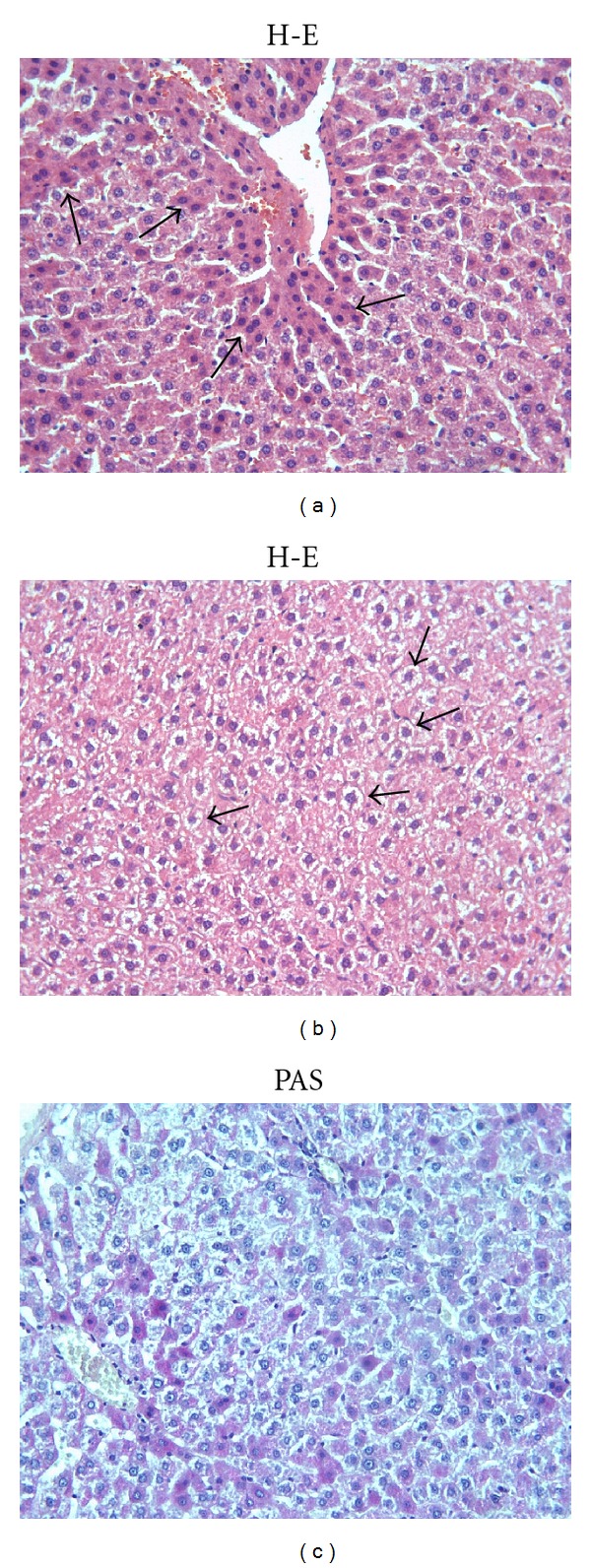
(a) Hepatocytes with eosinophilic cytoplasm (arrows) are observed in Mtx group. (b) Cellular swelling in hepatocytes (arrows) is noticed in Mtx group. (c) Marked reduction in glycogen content in Mtx group. X66.

**Figure 3:**
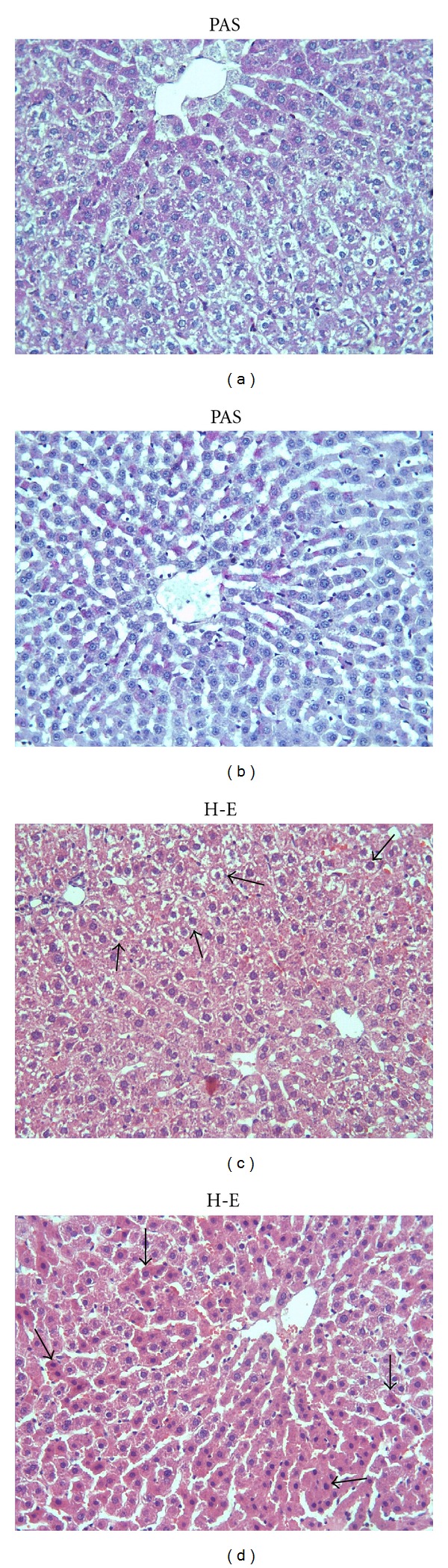
(a) Notice decreased glycogen storage in hepatocytes of Mtx + ML group. (b) View of glycogen storage in hepatocytes in ML + Mtx group. (c) The appearance of the swollen hepatocyte in Mtx + ML group (arrows). (d) Eosinophilic-stained hepatocytes are still observed (arrows) in ML + Mtx group. X66.

The control group and ML group showed a normal appearance of the liver cells as shown in Figures 1(a) and 1(b). In the Mtx group, major histological alterations were observed such as eosinophilic staining (Figure 2(a)) and swelling of hepatocytes (Figure 2(b)). Eosinophilic-stained hepatocytes were scattered randomly among the areas with normal morphology. The glycogen storage in hepatocytes was observed as decreased by PAS staining in the Mtx group as compared to controls. (Figure 2(c)). ML treatment did not completely ameliorate these lesions and milder degenerative alterations as loss of the glycogen content was still present (Figures 3(a) and 3(b)). Degenerative changes as swelling of hepatocytes in Mtx + ML group (Figure 3(c)), eosinophilic-stained hepatocytes in ML + Mtx group (Figure 3(d)) were evident; however, histopathological changes were not as extensive as in the Mtx group. Treatment with ML after Mtx injection and treatment with ML before Mtx injection were similar in term of microscopic damage.

Microscopic damage score for each group was determined and results were given in Table 2.

## 4. Discussion

The using of anticancer drugs is limited due to their acute toxic effects on some organs such as liver, kidney, testis, and heart [18–20]. It has been reported that Mtx-induced liver damage may occur by a high dose or by chronic application of methotrexate [6, 7]. Methotrexate may lead to liver hepatotoxicity, including steatosis, cholestasis, fibrosis, and cirrhosis [21]. The mechanisms of Mtx-hepatotoxicity can be related to its accumulation inside the cells in a polyglutamated form. This form causes decreasing folat levels and hepatotoxicity [22]. The other way; it is well known that oxidative stress plays a role in tissue damage caused by methotrexate [6, 23].

 In our study, methotrexate caused increasing in MDA and MPO levels. MDA, a stable metabolite of the free radical mediated lipid peroxidation cascade, is used widely as a marker of oxidative stress and lipid layers destroy [24]. As described above, methotrexate caused lipid peroxidations via a significant increase in MDA levels. Lipid peroxidation, mediated by oxygen-free radicals, is believed to be an important cause of destruction and damage to cell membranes and has been suggested to be a contributing factor to the development of methotrexate-mediated tissue damage [25]. Similarly, there were many studies about methotrexate-induced lipid peroxidations in liver tissue of rats though elevated MDA levels and these findings are in agreement with our results [3, 26, 27]. We and other reserchers thought that these effects of methotrexate may be due to its binding lipids in cell membrane [3, 28]. Also, it has determined that methotrexate leads to histological damage including eosinophilic-stained and swollen hepatocytes. The histological alterations may occur though methotrexate oxidative properties. These results are confirmed with other previous studies. In the current study, also MPO activity which is an index of inflammation increased in the methotrexate-treated group. Free radicals seem to trigger the accumulation of leukocytes in the tissues involved and thus aggravate tissue injury indirectly through activated neutrophils. It has been shown that activated neutrophils secrete enzymes (e.g., MPO, elastase, and proteases) and liberate oxygen radicals [29]. MPO, a member of the haem peroxidase-cyclooxygenase superfamily, is abundantly expressed in neutrophils and to a lesser extent in monocytes and certain type of macrophages. MPO plays a fundamental role in superoxide production [30]. The oxidative stress is an imbalance between lipid peroxdations and antioxidative system including GSH, a radical scavenger [31, 32]. In our study, there was no difference in GSH levels. One possible explanation of this finding was that GSH levels increased in chronic injury. Ozbek et al. [33] reported that GSH levels increased in chronic injury, whereas SOD and CAT enzyme activities are elevated in the acute phase of damage. We only give methotrexate to rats for one day, so GSH levels were not affected.

Montelukast, one of the selective reversible CysLT_1_ receptor antagonist, is used for the maintenance treatment of asthma and to relieve symptoms of seasonal allergies [34]. It is reported that montelukast can reduce eosinophilic inflammation in the airways [35–37]. Besides CysLT_1_ receptor antagonists or biosynthesis inhibitors ameliorate at ethanol-induced gastric mucosal damage [38] and wound healing [39, 40]. According to our results, montelukast treatment after Mtx injection reduced the MDA and MPO levels. This can be attributed to its antioxidant and anti-inflammatory capacity. In recent studies, it has been shown that montelukast has antioxidant effect [10–12]. Coskun et al. [41] and Cuciureanu et al. [8] reported that montelukast could reduce MDA and MPO levels as antioxidant. Our biochemical results suggested that montelukast treatment before methotrexate injection did not affect methotrexate-induced damage. However, in histological findings, treatment with montelukast after methotrexate injection and treatment with montelukast before methotrexate injection were similar in term of histological score. In addition, ML + Mtx group showed eosinophilic-stained hepatocytes. It is known that eosinophilic-stained cells show the starting irreversible damage in the tissue. On the other hand, there were degenerative changes as swelling of hepatocytes (reversble damage) in the Mtx + ML group. Whereas the histological scores of Mtx + ML and ML + Mtx groups are similar, Mtx + ML group showed reversbl damage.

Beside its antioxidant and anti-inflammatory effect, montelukast has other mechanisms of action through its decrease the severity hepatopathy. It is possible that montelukast could inhibit the chloride conductance in hepatocytes. It is also known that LTD_4, _a member of LTs, activates a chloride conductance in hepatocytes, and ion channel activation is associated with cytotoxicity [42].

## 5. Conclusion

The present study demonstrates that montelukast treatment after methotrexate injection reduces the oxidative damage in the liver tissue. These therapeutic effects can be attributed to its action on oxidant-antioxidant systems and inflammation process. Although, further experimental and clinical studies are required to confirm these findings before its clinical applications against liver injury.

## Figures and Tables

**Table 1:** The levels of biochemical parameters of all groups.

Parameters	Control	ML	Mtx	Mtx + ML	ML + Mtx
MDA (nmol/g tissue)	937.5 ± 71.03	872.3 ± 120.3	1669.9 ± 129.7^a^	443.1 ± 32.7^b,c^	1500 ± 80.9
GSH (nmol/g tissue)	1937 ± 193.7	2116.3 ± 189.1	2185.9 ± 321.6	1847.0 ± 111.1	1621 ± 219.6
MPO (U/g protein)	58.3 ± 7.9	70.07 ± 11.7	201.9 ± 15.5^a^	80.7 ± 10.7^b^	154.9 ± 29.8

^a^ *P* < 0.05, when compared to the control and ML groups.

^b^ *P* < 0.05, when compared to the Mtx group.

^c^ *P* < 0.05, when compared to the other groups.

**Table 2:** Comparison of the effect of ML on microscopic damage caused by Mtx in liver.

Parameters	Control	ML	Mtx	Mtx + ML	ML + Mtx
Microscopic damage	0.3 ± 0.5	0.8 ± 0.9	4.7 ± 0.9^a^	3.3 ± 0.5^b,c^	3.2 ± 0.7^b^

^a^Significantly increased when compared with control group, *P* = 0.001.

^b^Significantly decreased when compared with Mtx group, *P* = 0.005 and *P* = 0.01.

^c^No significance when compared with ML + Mtx, *P* = 0.8.
